# Analysis of cytotoxic T lymphocytes from a patient with hepatocellular carcinoma who showed a clinical response to vaccination with a glypican-3-derived peptide

**DOI:** 10.3892/ijo.2013.2044

**Published:** 2013-07-31

**Authors:** YOSHITAKA TADA, TOSHIAKI YOSHIKAWA, MANAMI SHIMOMURA, YU SAWADA, MAYUKO SAKAI, HIROFUMI SHIRAKAWA, DAISUKE NOBUOKA, TETSUYA NAKATSURA

**Affiliations:** 1 Division of Cancer Immunotherapy, Research Center for Innovative Oncology, National Cancer Center Hospital East, Kashiwa, Chiba 277-8577;; 2 Research Institute for Biomedical Sciences, Tokyo University of Science, Noda, Chiba 278-0022, Japan

**Keywords:** glypican-3, peptide vaccine, CTL clone

## Abstract

Glypican-3 (GPC3), which is a carcinoembryonic antigen, is overexpressed in human hepatocellular carcinoma (HCC). Previously, we performed a phase I clinical trial of GPC3-derived peptide vaccination in patients with advanced HCC, and reported that GPC3 peptide vaccination is safe and has clinical efficacy. Moreover, we proposed that a peptide-specific CTL response is a predictive marker of overall survival in patients with HCC who receive peptide vaccination. In this study, we established GPC3-derived peptide-specific CTL clones from the PBMCs of an HLA-A ^*^ 02:07-positive patient with HCC who was vaccinated with an HLA-A2-restricted GPC3 peptide vaccine and showed a clinical response in the phase I clinical trial. Established CTL clones were analyzed using the IFN-γ ELISPOT assay and a cytotoxicity assay. GPC3 peptide-specific CTL clones were established successfully from the PBMCs of the patient. One CTL clone showed cytotoxicity against cancer cell lines that expressed endogenously the GPC3 peptide. The results suggest that CTLs have high avidity, and that natural antigen-specific killing activity against tumor cells can be induced in a patient with HCC who shows a clinical response to vaccination with the GPC3 _144–152_ peptide.

## Introduction

Primary liver cancer, which is frequently hepatocellular carcinoma (HCC), is the sixth most common cancer and third most frequent cause of cancer-related death worldwide, and it is becoming more prevalent not only in East Asia, South-East Asia, and Africa but also in Western countries ( 1 – 3 ) . Recently, the multikinase inhibitor sorafenib was demonstrated to prolong overall survival (OS) in patients with advanced HCC, and it has become the standard drug for first-line systemic treatment ( 4 – 6 ) . However, based on the Response Evaluation Criteria in Solid Tumors (RECIST), the response rate for sorafenib is rather low, and the incidence of adverse events is relatively high, especially in elderly patients ( 7 ) . Therefore, the generation of a novel effective therapy for HCC is a priority.

Immunotherapy is an attractive option for treating HCC. Many of the tumor antigens associated with HCC are potential candidates for peptide vaccines ( 8 , 9 ) . The carcinoembryonic antigen Glypican-3 (GPC3), which is a 65-kDa protein of 580 amino acids, belongs to the family of glycosyl-phosphatidylinositol (GPI)-anchored heparan sulfate proteoglycans (HSPG) ( 10 , 11 ) . GPC3 is specifically overexpressed in HCC (72–81% of cases) and correlates with poor prognosis ( 12 – 16 ) . This suggests that GPC3 is an ideal target for anti-HCC immunotherapy.

We have previously demonstrated the antigenicity of GPC3, and that the HLA-A ^*^ 24:02-restricted GPC3 _298–306_ (EYILSLEEL) peptide and the HLA-A ^*^ 02:01-restricted GPC3 _144–152_ (FVGEFFTDV) peptide can induce GPC3-reactive CTLs without inducing autoimmunity ( 17 – 21 ) .

HLA-A2 is the most frequent HLA-A type in all ethnic groups ( 22 ) . HLA-A2 is also expressed in about 40% of Japanese persons ( 23 , 24 ) and in about 50% of Caucasians ( 25 ) . Among Caucasians, >90% of HLA-A2-positive individuals carry the HLA-A ^*^ 02:01 allele ( 25 ) , whereas among the Japanese, there are multiple common and well-documented (CWD) allelic variants, including HLA-A ^*^ 02:01, HLA-A ^*^ 02:06 and HLA-A ^*^ 02:07 ( 26 ) . The frequencies of the HLA-A ^*^ 02:01, HLA-A ^*^ 02:06 and HLA-A ^*^ 02:07 alleles in the Japanese population are 19, 14 and 7%, respectively ( 26 ) . Therefore, we confirmed that the HLA-A ^*^ 02:01-restricted GPC3 _144–152_ (FVGEFFTDV) peptide could also bind to HLA-A ^*^ 02:06 and HLA-A ^*^ 02:07 using a binding assay (unpublished data).

On the basis of these results, we conducted a phase I clinical trial of a GPC3-derived peptide vaccine in 33 patients with advanced HCC. The HLA-A ^*^ 24:02-restricted GPC3 _298–306_ peptide was used for HLA-A ^*^ 24:02-positive patients and the HLA-A ^*^ 02:01-restricted GPC3 _144–152_ peptide was used for HLA-A ^*^ 02:01, HLA-A ^*^ 02:06 and HLA-A ^*^ 02:07-positive patients. We found that GPC3 vaccination was well-tolerated, and that the GPC3 peptide vaccine induced a GPC3-specific CTL response in almost all of the patients ( 27 – 30 ) . Moreover, the vaccination-induced GPC3-specific CTL response correlated with overall survival (OS); the OS was significantly longer in patients with high GPC3-specific CTL frequencies than in those with low GPC3-specific CTL frequencies ( 27 ) . In terms of clinical responses, one patient showed a partial response (PR) and 19 patients showed stable disease 2 months after initiation of treatment. One patient with HCC who showed a PR was HLA-A ^*^ 02:07-positive. In addition, several HLA-A ^*^ 02:01-restricted GPC3 peptide-specific CTL clones with cytotoxic activities against GPC3 were established from the peripheral blood mononuclear cells (PBMCs) of patients vaccinated in this trial ( 27 ) .

The aims of the present study were: i) to establish GPC3-derived, peptide-specific CTL clones from the PBMCs of an HLA-A ^*^ 02:07-positive patient with HCC who showed a PR in the phase I clinical trial; and ii) to analyze the functions of these CTL clones.

## Materials and methods

### Ethics information

This study was approved by the Ethics Committee of the National Cancer Center and conformed to the ethical guidelines of the 1975 Declaration of Helsinki. All the patients gave written informed consent before entering the study at the National Cancer Center Hospital East (Chiba, Japan). The trial has been registered with the University Hospital Medical Information Network Clinical Trials Registry (UMIN-CTR no. 000001395).

### PBMCs collection

Peripheral blood samples were obtained pre- and post-vaccination from the patient with HCC who was HLA-A ^*^ 02:07-positive. Post-vaccination, blood samples were collected from the patient every 2 weeks. The GMP-grade peptide GPC3 _144–152_ (FVGEFFTDV) (American Peptide Co., Sunnyvale, CA, USA) was emulsified in IFA (Montanide ISA-51 VG; SEPPIC, Paris, France) and injected intradermally at 30 mg/body three times at 14-day intervals ( 27 , 28 ) . PBMCs were isolated by density centrifugation using Ficoll-Hypaque (Pharmacia, Uppsala, Sweden) and frozen in liquid nitrogen until use.

### Cell lines

The human lung cancer cell line 1–87 (GPC3 ^−^ , HLA-A ^*^ 02:07 ^+^ /A ^*^ 11:01 ^+^ ) and hepatitis B virus (HBV)-integrated human hepatocellular carcinoma cell line JHH-7 (GPC3 ^+^ , HLA-A ^*^ 24:02 ^+^ /A ^*^ 31:01 ^+^ ) were conserved in our laboratory and cultured in Dulbecco’s modified Eagle’s medium (DMEM; Sigma Chemical Company, St. Louis, MO, USA) that was supplemented with 10% heat-inactivated fetal bovine serum (FBS; Gibco, Carlsbad, CA, USA).

### Plasmids and transfection

The expression vectors pcDNA3.1 (Invitrogen, Carlsbad, CA, USA) and pcDNA3.1 that contained the HLA-A ^*^ 02:07 cDNA were used for the transfection experiments. The pcDNA3.1 construct that contained HLA-A ^*^ 02:07 was kindly provided by Dr Ryo Abe (Tokyo University of Science, Chiba, Japan). The JHH-7/HLA-A ^*^ 02:07 cell line was obtained by transfection of JHH-7 cells with the expression vector using FuGENE HD (Roche Applied Science, Mannheim, Germany). JHH-7/mock and JHH-7/HLA-A ^*^ 02:07 cells were cultured in DMEM that was supplemented with 10% heat-inactivated FBS and 1 mg/ml G418 (Calbiochem, Darmstadt, Germany).

### Induction of GPC3 _144–152_ peptide-specific CTLs from PBMCs

The PBMCs were cultured (2×10 ^6^ cells/well) with the GPC3 _144–152_ peptide in RPMI-1640 (Sigma Chemical Company) that was supplemented with 10% heat-inactivated FBS, 100 IU/ml recombinant human IL-2 (Nipro, Osaka, Japan), and 10 ng/ml recombinant human IL-15 (PeproTech Inc, Rocky Hill, NJ, USA) for 14 days.

### CD107a staining and flow cytometry analysis

CD8 ^+^ T cells were isolated using human CD8 microbeads (Miltenyi Biotec, Bergisch Gladbach, Germany) from PBMCs that were stimulated with the GPC3 _144–152_ peptide for 14 days. The CD8 ^+^ T cells were incubated with GPC3 _144–152_ -pulsed or HIV _19–27_ -pulsed 1–87 cells at a ratio of 2:1 for 3.5 h at 37°C. CD107a-specific antibodies (BD Biosciences, San Jose, CA, USA) were included in the mixture during the incubation period.

### Generation of CTL clones

CD8 ^+^ CD107a ^+^ cells were sorted using a FACSAria cell sorter (BD Biosciences). Sorted CTLs were stimulated and the CTL clones were established as previously described ( 28 ) .

### Cytotoxicity assay

Cytotoxic capacity was analyzed with the Terascan VPC system (Minerva Tech, Tokyo, Japan). The CTL clone was used as the effector cell type. Target cells were labeled in calcein-AM solution for 30 min at 37°C. The labeled cells were then co-cultured with the effector cells for 4–6 h. Fluorescence intensity was measured before and after the culture period, and specific cytotoxic activity was calculated as previously described ( 28 ) .

### IFN-γ ELISPOT assay

Specific secretion of IFN-γ from human CTLs in response to stimulator cells was assayed using the IFN-γ ELISPOT kit (BD Biosciences), according to the manufacturer’s instructions. Stimulator cells were pulsed with or without peptide for 1.5 h at room temperature and then washed three times. Responder cells were incubated with stimulator cells for 20 h. The resulting spots were counted using an ELIPHOTO counter (Minerva Tech).

### Determination of recognition efficiency

Calcein-AM-labeled target cells were pulsed with various concentrations of peptide, starting at 10 ^−6^ M and decreasing in log steps to 10 ^−14^ M. The CTL clones were incubated with the target cells at an effector:target (E/T) ratio of 10:1 for 4 h. The recognition efficiencies of the CTL clones were defined as previously described ( 28 ) .

### RNA interference

Human GPC3-specific siRNAs were chemically synthesized as double-strand RNA (Invitrogen). A non-silencing siRNA, AllStras Neg. Control siRNA, was obtained from Qiagen (Valencia, CA, USA). The following GPC3-specific siRNA sequences were used: GPC3-siRNA (#4149), 5′-UUAUCAUUCCAUCACCAGAGCCUCC-3′; GPC3-siRNA (#4150), 5′-GGAGGCUCUGGUGAUGGAAU GAUAA-3′: and GPC3-siRNA (#4151), 5′-UAUAGAUGACUG GAAACAGGCUGUC-3′. Synthetic siRNA duplexes were transfected using Lipofectamine RNAiMAX (Invitrogen), according to the manufacturer’s protocols.

### RT-PCR

Using the TRIzol reagent (Invitrogen), we extracted total cellular RNA from untreated or siRNA (GPC3-siRNA or negative-siRNA)-treated JHH-7/HLA-A ^*^ 02:07. cDNA was synthesized using the PrimeScript II 1st Strand cDNA Synthesis kit (Takara, Kyoto, Japan) according to the manufacturer’s instructions. The cDNA was added to a reaction mix that contained 10X Ex Taq Buffer (Takara), 2.5 mM dNTP mixture (Takara), 5 units Ex Taq (Takara), and 10 μM of the *GPC3* - or *β-actin* -specific PCR primers. The following primer sequences (sense and antisense, respectively) were used: for *GPC3* , 5′-AGCCAAAAGGCAGCAAGGAA-3′ and 5′-AAGA AGAAGCACACCACCGA-3′; and for *β-actin* , 5′-CCTCGCCT TTGCCGATCC-3′ and 5′-GGATCTTCATGAGGTAGTC AGTC-3′. PCR was performed using the 96-well Gene Amp PCR System 9700 (Applied Biosystems, Carlsbad, CA, USA). PCR was performed for 20 cycles of 98°C for 10 sec, 64°C for 30 sec and 72°C for 30 sec, followed by a step of 72°C for 10 sec.

### Sequence analysis of TCR-β gene

Using the TRIzol reagent (Invitrogen), total cellular RNA was extracted from established CTL clones. The cDNA of the *TCR-β* gene was synthesized using the PrimeScript II 1st Strand cDNA Synthesis Kit (Takara) according to the manufacturer’s instructions, with the modification that we used 200 nM of the primer specific for the TCR-β chain constant region. The cDNA products were subjected to 2-step PCR, as previously described by Yukie Tanaka-Harada ( 35 , 36 ) , and the PCR products were purified and sequenced in the Applied Biosystems 3500 Genetic Analyzer (Applied Biosystems). The *TCR-β* variable ( *TRBV* ) gene, *TCR-β* joining ( *TRBJ* ) gene, *TCR-β* diversity ( *TRBD* ) alleles, and complementarity-determining region 3 ( *CDR3* ) sequences were identified using the IMGT databases ( http://www.imgt.org/ ).

## Results

### GPC3 _144–152_ peptide-specific CTLs in the peripheral blood of the patient exert a clinical effect

**Figure 1.**
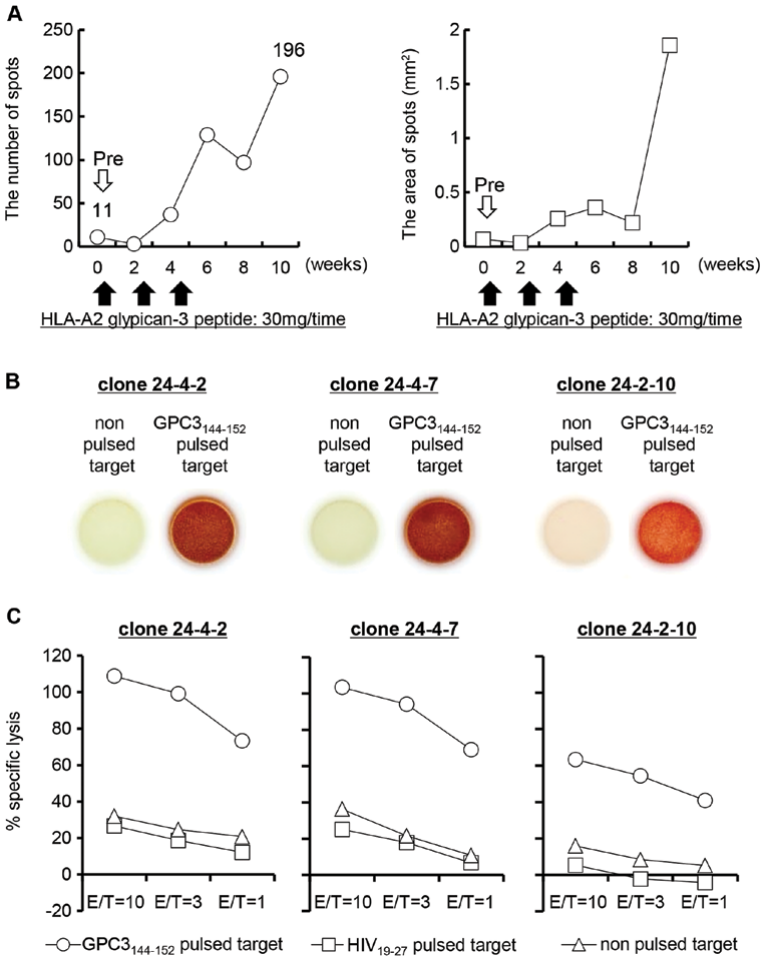
GPC3 peptide-specific CTL clones established from the PBMCs of a patient following GPC3 peptide vaccination. (A) Changes in the frequencies of GPC3 _144–152_ peptide-specific CTLs before and after vaccination in a patient who showed a PR post-vaccination. Changes in the GPC3 peptide-specific CTLs are observed as differences in the number (left) and the area (right) of spots in an *ex vivo* IFN-γ ELISPOT assay. (B) Results of the IFN-γ ELISPOT assay against peptide-pulsed target. HLA-A ^*^ 02:07 ^+^ cancer cell line 1–87 was used as the target. The target was pulsed with the GPC3 _144–152_ peptide. A non-pulsed target was used as the negative control. The ratio of effector cells to target cells (E/T) is 1. (C) Results of the cytotoxicity assay against peptide-pulsed target. The 1–87 cells were used as the target. Non-pulsed and HIV _19–27_ peptide-pulsed targets were used as negative controls. E/Ts are 10, 3 and 1, respectively. A representative of three experiments is shown.

We analyzed the immune responses of the patient who showed a PR following GPC3 _144–152_ peptide vaccination. In this patient, the supraclavicular lymph node metastases markedly regressed, two liver tumors disappeared, and the thoracic bone metastasis showed necrosis after the third vaccination ( 27 ) . The levels of DCP decreased in the patients over the 2-month period. We evaluated the GPC3 _144–152_ -specific immune responses in the peripheral blood using the *ex vivo* IFN-γ ELISPOT assay. For the HLA-A ^*^ 02:07-positive patient with advanced HCC, the number and area of the spots increased after two rounds of vaccination, as compared with the pre-vaccination values, and the peak values were noted 10 weeks after the start of the treatment ( Fig. 1A ).

### Establishment of GPC3 _144–152_ -specific CTL clones from the PBMCs of the patient

To investigate the ability of the GPC3 _144–152_ -specific CTLs induced by peptide vaccination to recognize antigen, we established CTL clones from the PBMCs of this patient 10 weeks after the start of treatment. The PBMCs were stimulated with the GPC3 _144–152_ peptide *in vitro* for 14 days. CD8 ^+^ T cells were isolated from the stimulated PBMCs, and then incubated with peptide-pulsed 1–87 cells. CD8 ^+^ CD107a ^+^ cells that reacted with the GPC3 _144–152_ -pulsed 1–87 cells were sorted to the single-cell level. Thus, we established GPC3 _144–152_ peptide-specific CTL clones.

Three established CTL clones were analyzed for function using the IFN-γ ELISPOT assay and cytotoxicity assay. All of the CTL clones released IFN-γ in response to the GPC3 _144–152_ -pulsed 1–87 cells, but not in response to non-pulsed 1–87 cells ( Fig. 1B ). Moreover, these CTL clones showed cytotoxicity against GPC3 _144–152_ -pulsed 1–87 cells, but not against non-pulsed or HIV19-27-pulsed 1–87 cells ( Fig. 1C ). These results indicate that the CTL clones 24-4-2, 24-4-7 and 24-2-10 have specificity for the GPC3 _144–152_ peptide.

### Functional avidity of the GPC3 _144–152_ -specific CTL clones

**Figure 2.**
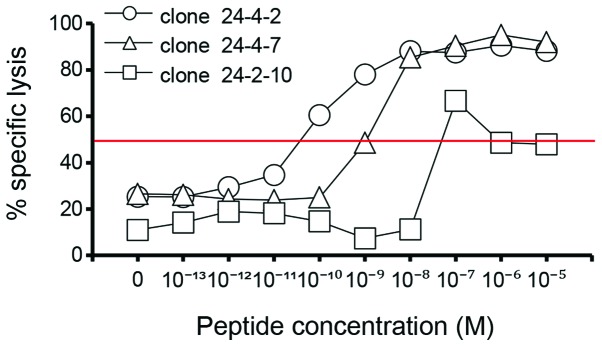
GPC3 _144–152_ peptide-specific avidity of the established CTL clones. The established CTL clones were tested for avidity using 1–87 cells that were pulsed with various concentrations of the GPC3 _144–152_ peptide. The peptide concentration at which the curve crossed the 50% cytotoxicity mark was defined as the recognition efficiency of that clone. E/T is 10. A representative of three experiments is shown.

We evaluated the cytotoxicity profiles of the CTL clones for 1–87 cells pulsed with a decreasing concentration series (from 10 ^−6^ to 10 ^−14^ M) of the GPC3 _144–152_ peptide. The peptide concentration at which the curve reached 50% cytotoxicity was defined as the recognition efficiency of the clone. The recognition efficiencies of CTL clones 24-4-2, 24-4-7 and 24-2-10 were 10 ^−11^ , 10 ^−9^ and 10 ^−8^ M, respectively ( Fig. 2 ). This result suggests that CTL clone 24-4-2 has a higher avidity than the other two clones and, conversely, that CTL clone 24-2-10 has a lower avidity than the other two clones.

### A GPC3 _144–152_ -specific CTL clone recognizes cancer cells that endogenously express GPC3

**Figure 3.**
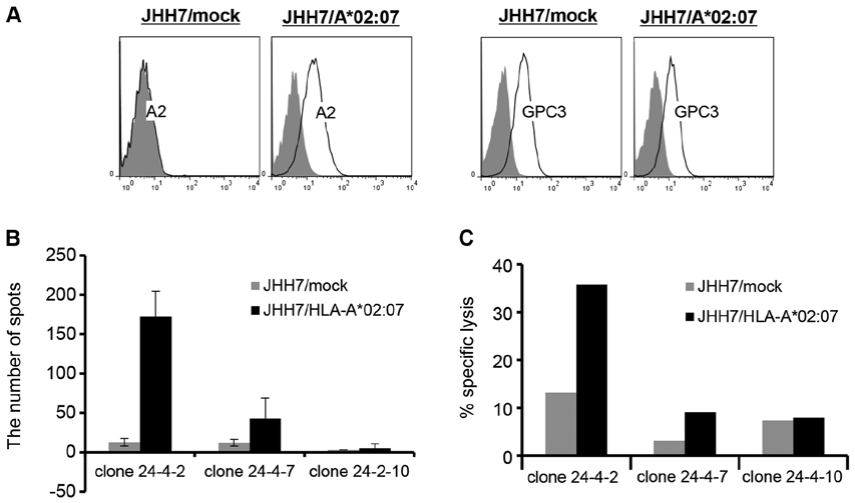
Recognition of GPC3 ^+^ cancer cells by the established CTL clones. (A) Expression of HLA-A2 (left panel) and GPC3 (right panel) on established GPC3 ^+^ HLA-A ^*^ 02:07 ^+^ cancer cells and control cells. (B) Results of the IFN-γ ELISPOT assay for the GPC3 ^+^ cancer cell line. The HLA-A ^*^ 02:07-overexpressing GPC3 ^+^ cancer cell line, JHH7/HLA-A ^*^ 02:07, was established and used as the target. JHH7/mock cells were used as the negative control. E/T ratio, 1. Data are presented as mean ± SD of three independent batches. (C) Results of the assay for cytotoxicity against the GPC3 ^+^ cancer cell line. JHH7/HLA-A ^*^ 02:07 cells were used as the target. JHH7/mock cells were used as the negative control. E/T is 3. A representative of three experiments is shown.

Next, we tested the reactivities of these CTL clones against cancer cell lines that expressed GPC3 and HLA-A ^*^ 02:07. We used the JHH-7/mock (GPC3 ^+^ , HLA-A ^*^ 02:07-) and JHH-7/HLA-A ^*^ 02:07 (GPC3 ^+^ , HLA-A ^*^ 02:07 ^+^ ) transfectants as the target cells ( Fig. 3A ). The CTL clone 24-4-2 (with high avidity) produced IFN-γ and was cytotoxic for JHH-7/HLA-A ^*^ 02:07 cells but not for JHH-7/mock cells ( Fig. 3B and C ). The other clones did not produce IFN-γ and did not exhibit cytotoxicity for the two target cell lines. These results suggest that only high-avidity CTLs recognize cancer cells that express GPC3 peptide endogenously.

### CTL clone 24-4-2 shows specificity for GPC3

**Figure 4.**
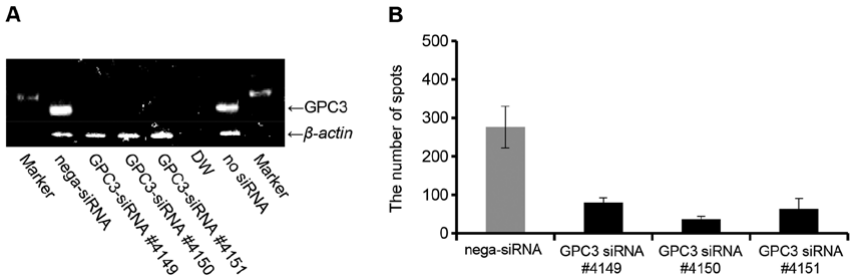
GPC3 specificity of CTL clone 24-4-2. (A) GPC3 expression levels on JHH7/HLA-A ^*^ 02:07 cells treated with GPC3-siRNA or negative (nega)-siRNA for 48 h, as determined by RT-PCR. (B) Results of the IFN-γ ELISPOT assay for JHH7/HLA-A ^*^ 02:07 cells treated with GPC3-siRNA or nega-siRNA. E/T is 1. Data are presented as mean ± SD of three independent batches.

To ascertain the GPC3 antigen-specific response of CTL clone 24-4-2, we created a GPC3 knockdown via siRNA treatment of the JHH-7/HLA-A ^*^ 02:07 cells. GPC3 expression by the JHH-7/HLA-A ^*^ 02:07 cells was clearly decreased by the GPC3-siRNA, as assessed by RT-PCR ( Fig. 4A ). We examined the IFN-γ production levels of CTL clone 24-4-2 against JHH-7/HLA-A ^*^ 02:07 cells treated with GPC3-siRNA. IFN-γ production by CTL clone 24-4-2 was significantly decreased by the GPC3-siRNA ( Fig. 4B ). These results indicate that the HLA-A2-restricted GPC3 _144–152_ peptide is processed naturally by cancer cells, and that both HLA-A ^*^ 02:07 and HLA-A ^*^ 02:01 can present the GPC3 _144–152_ peptide on the surfaces of cancer cells.

### Established CTL clones have different sets of TCR-β alleles

We analyzed the *TCR-β* gene sequences of the established CTL clones. The *TRBV* , *TRBJ* and *TRBD* alleles were identified using the IMGT databases. Thus, we identified the *TRBV* , *TRBD* and *TRBJ* alleles of the CTL clones ( Table I ). Each of the established CTL clones had different allele sets.

### CTL clone 24-4-2 is subject to HLA-A ^*^ 02:07 restriction

**Figure 5.**
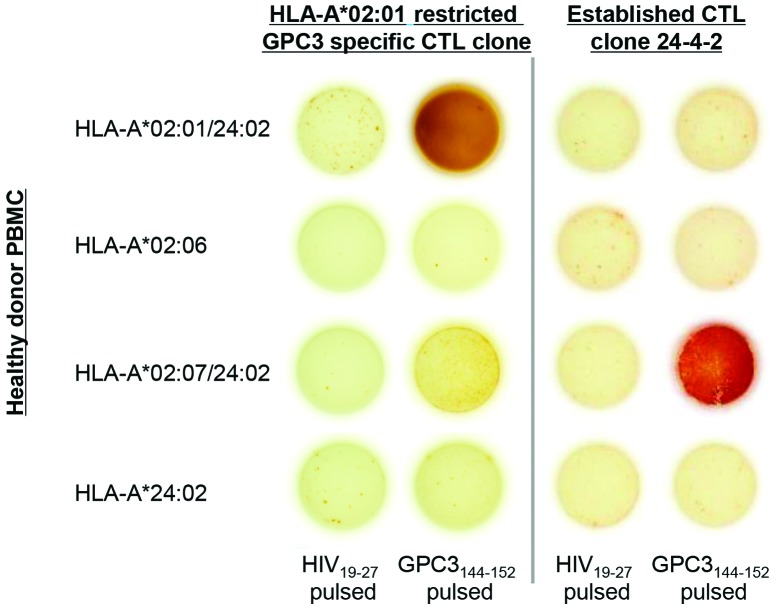
CTL clone 24-4-2 shows HLA-A ^*^ 02:07 restriction. Results of the IFN-γ ELISPOT assay for healthy donor PBMCs with HLA-A2. The established CTL clone 24-4-2 and the HLA-A ^*^ 02:01-restricted, GPC3-specific CTL clone were used as effectors. E/T is 0.2. A representative of two experiments is shown.

We investigated whether CTL clone 24-4-2 recognized the GPC3 _144–152_ peptide-HLA-A ^*^ 02:01 complex and the GPC3 _144–152_ peptide-HLA-A ^*^ 02:06 complex, as well as the GPC3 _144–152_ peptide-HLA-A ^*^ 02:07 complex. Healthy donor PBMCs with HLA-A ^*^ 02:01, HLA-A ^*^ 02:06, HLA-A ^*^ 02:07 and HLA-A ^*^ 24:02 were used as the targets, and an HLA-A ^*^ 02:01-restricted, GPC3-specific CTL clone, which is a previously established CTL clone ( 26 ) , was used as the control. The HLA-A ^*^ 02:01-restricted CTL clone recognized only the GPC3 _144–152_ peptide-HLA-A ^*^ 02:01 complex, and CTL clone 24-4-2 recognized only the GPC3 _144–152_ peptide-HLA-A ^*^ 02:07 complex ( Fig. 5 ). These outcomes indicate that CTL clone 24-4-2 has HLA-A ^*^ 02:07 restriction.

## Discussion

Clinical trials of peptide-based vaccines are underway in several parts of the world. However, the monitoring of individual CTL post-vaccination has scarcely been reported in immunotherapy trials. In the present study, we established HLA-A ^*^ 02:07 ^+^ GPC3 _144–152_ -specific CTL clones from the PBMCs of a patient who showed a PR following GPC3-derived peptide vaccination and we performed functional analyses against established CTL clones.

This patient showed an increase in the number of CTLs specific for the GPC3-derived peptide in the peripheral blood after vaccination ( Fig. 1A ) ( 27 , 28 ) . Ten weeks after the start of treatment, the GPC3 _144–152_ -specific CTL counts had increased approximately 18-fold, as compared with the pre-vaccination counts. In this case, analysis of the established CTL clones after vaccination could lend support to the notion that the vaccine-induced CTLs exert an antitumor effect, since few GPC3 _144–152_ -specific CTLs were detected before vaccination.

In the present study, we confirmed that GPC3 _144–152_ -specific CTL clones are cytotoxic for both GPC3 _144–152_ -pulsed 1–87 cells and JHH-7/HLA-A ^*^ 02:07 cells that express GPC3 peptide endogenously. Confirming that the GPC3 peptide-specific CTL clones kill cancer cells that express endogenously the antigen peptide is important because antigen-derived and CTL-inducible peptides are not necessarily presented by cancer cells that endogenously express the antigen ( 31 – 33 ) . Three established CTL clones showed cytotoxic activities related to their avidity for GPC3 _144–152_ -pulsed 1–87 cells and JHH-7/HLA-A ^*^ 02:07 cells that expressed the GPC3 peptide endogenously. These results show that although CTLs with different avidity can be isolated, only those CTLs with high avidity can kill cancer cells that express the antigen peptide endogenously. Several investigators have demonstrated a correlation between T-cell avidity and target recognition by T-cell populations that recognize murine tumor models and human cancers ( 34 ) . Our results strongly support this observation.

The TCR usage of antigen-specific T cells is thought to be influenced by the affinity of the TCR for the antigen peptide-HLA class I complex. Several studies on the TCR usage of tumor-associated antigen (TAA)-specific T cells have used the *TRBV* gene family ( 35 – 41 ) . These studies mainly analyzed the frequencies of TAA tetramer positive CD8 ^+^ T cells. Although it is important to examine quantitative aspects, such as the frequencies of TAA tetramer positive CD8 ^+^ T cells, the cytotoxicity of these T cells against cancer cells that express the TAA peptide endogenously cannot be confirmed. Moreover, GPC3 dextramer positive CD8 ^+^ T cells were not detected in the PBMCs of the patients with HCC before GPC3 peptide vaccination ( 27 , 28 ) . To analyze biased usage of the *TCR* gene of GPC3 dextramer positive CD8 ^+^ T cells in the patients with HCC before and after GPC3 peptide vaccination, a new detection system with greater sensitivity *ex vivo* will be required. In the present study, we analyzed the *TCR-β* genes of the established GPC3 _144–152_ -specific CTL clones, to confirm that these CTL clones have different TCRs. Our experiments show that the established CTL clones have different TCR-β-chain allele sets, i.e., *TRBV* , *TRBD* and *TRBJ* alleles ( Table I ), and different CDR3 sequences (data not shown). These results suggest that various GPC3-specific CTLs are induced by GPC3 _144–152_ peptide vaccination.

A ^*^ HLA-A ^*^ 02:07 differs from HLA-A ^*^ 02:01 by a single non-conservative change (Y to C) at residue 99. X-ray crystallographic data have identified position 99 as one of the residues forming the D secondary pocket, which engages the residue at position 3 on peptide ligands ( 42 – 44 ) . Although hHLA-A ^*^ 02:07 was originally not included in the HLA-A2 supertype, cross-reactivity between HLA-A ^*^ 02:07 and other A2 subtypes was detected at the functional level ( 44 , 45 ) . Moreover, this HLA molecule indeed binds a subset of the peptide repertoire bound by other A2 subtypes ( 44 ) . For these reasons, HLA-A ^*^ 02:07 should also be included in the A2 supertype ( 46 ) . Ito *et al* ( 47 ) and Nonaka *et al* ( 48 ) reported that an HLA-A2-restricted CTL line established from the tumor-infiltrating lymphocytes (TIL) of an HLA-A ^*^ 02:07-positive patient showed significant cytotoxicities for HLA-A ^*^ 02:01-, HLA-A ^*^ 02:06- and HLA-A ^*^ 02:07-positive cancer cells. Therefore, we examined whether the GPC3 _144–152_ -specific CTL clone 24-4-2, which was established from the PBMCs of an HLA-A ^*^ 02:07- positive patient with HCC, could recognize HLA A-A ^*^ 02:01 or HLA-A ^*^ 02:06. However, this CTL clone failed to recognize HLA-A ^*^ 02:01 or HLA-A ^*^ 02:06.

We have reported previously on the detection via immunohistochemical staining of massive infiltration of CD8-positive T cells into the remaining liver tumor of this patient ( 27 ) . It was difficult to confirm that these tumor-infiltrating CD8 ^+^ T cells have specificity for GPC3. Currently, we are conducting clinical testing of liver biopsies taken before and after GPC3 peptide vaccination of patients with advanced HCC. Our aim is to reveal the GPC3 peptide-specific immune responses induced by the GPC3-derived peptide vaccine in both the peripheral blood and the tumor. We are analyzing the *TCR* gene sequences of CD8 or GPC3 dextramer positive T cells in both the peripheral blood and tumor. Already in this trial, a remarkable clinical effect has been observed for an HLA-A ^*^ 02:07-positive patient with HCC who received GPC3 _144–152_ peptide vaccination ( 49 ) .

HLA-A ^*^ 02:07 is present in the populations of East Asia, South-East Asia (7%), and northern India (11.5%) ( 26 , 50 – 52 ) . In southern China, the frequency of the HLA-A ^*^ 02:07 allele is reported to be even higher than the frequency of the HLA-A ^*^ 02:01 allele ( 53 , 54 ) . In addition, about 75% of liver cancer cases occur in South-East Asia, including China, Hong Kong, Taiwan, Korea, India and Japan ( 55 ) . Taking together these previous reports and our results, it appears that HLA-A ^*^ 02:07-positive patients with HCC are good candidates for GPC3 _144–152_ peptide vaccination. Further studies will be necessary to prove the clinical efficacy of GPC3 peptide vaccination for advanced HCC.

In conclusion, we present substantial evidence that GPC3 _144–152_ -specific CTLs with different TCR allele sets that are induced in patients with HCC who show a PR following GPC3 _144–152_ peptide vaccination indicate not only high avidity but also natural antigen-specific killing activity against tumor cells.

## Figures and Tables

**Table I.** TCR-β chain sequencing for established CTL clones.

No.	TRBV	TRBJ	TRBD
Clone 24-4-2	18 ^ * ^ 01	1–2 ^ * ^ 01	1 ^ * ^ 01
Clone 24-4-7	7-3 ^ * ^ 01	2–7 ^ * ^ 01	1 ^ * ^ 01
Clone 24-2-10	7-6 ^ * ^ 01	2-1 ^ * ^ 01	2 ^ * ^ 01
